# Spatial Variation of Airborne Pollen Concentrations Locally around Brussels City, Belgium, during a Field Campaign in 2022–2023, Using the Automatic Sensor Beenose

**DOI:** 10.3390/s24123731

**Published:** 2024-06-08

**Authors:** Jean-Baptiste Renard, Houssam El Azari, Johann Lauthier, Jérémy Surcin

**Affiliations:** 1 LPC2E-CNRS, 3A Avenue de la Recherche Scientifique, CEDEX 2, 45071 Orleans, France; houssam.elazari@lifyair.com; 2 LIFY-AIR, Le LAB’O, 1 Avenue du Champ de Mars, 45100 Orleans, France; johann.lauthier@lifyair.com; 3 Pollutrack, 5 rue Lespagnol, 75020 Paris, France; jeremysurcin.pollutrack@protonmail.com

**Keywords:** automatic pollen monitoring, Beenose sensor, outdoor campaign

## Abstract

As a growing part of the world population is suffering from pollen-induced allergies, increasing the number of pollen monitoring stations and developing new dedicated measurement networks has become a necessity. To this purpose, Beenose, a new automatic and relatively low-cost sensor, was developed to characterize and quantify the pollinic content of the air using multiangle light scattering. A field campaign was conducted at four locations around Brussels, Belgium, during summer 2022 and winter–spring 2023. First, the consistency was assessed between the automatic sensor and a collocated reference Hirst-type trap deployed at Ixelles, south-east of Brussels. Daily average total pollen concentrations provided by the two instruments showed a mean error of about 15%. Daily average pollen concentrations were also checked for a selection of pollen species and revealed Pearson and Spearman correlation coefficients ranging from 0.71 to 0.93. Subsequently, a study on the spatial variability of the pollen content around Brussels was conducted with Beenose sensors. The temporal evolution of daily average total pollen concentrations recorded at four sites were compared and showed strong variations from one location to another, up to a factor 10 over no more than a few kilometers apart. This variation is a consequence of multiple factors such as the local vegetation, the wind directions, the altitude of the measurement station, and the topology of the city. It is therefore highly necessary to multiply the number of measurement stations per city for a better evaluation of human exposure to pollen allergens and for more enhanced pollen allergy management.

## 1. Introduction

An increasing part of the population suffers from pollen-induced allergies, with symptoms such as conjunctivitis, rhinitis, or asthma. At least 40% of the European population is affected by these allergies [1] and, in the United States, a high prevalence (up to 26%) of positive skin test responses to ragweed and grass is reported in the population [2]. Airborne pollen particles have a significant impact on human health [3,4], entailing significant costs to the health system and to society as a whole [5,6]. In addition, climate change and ambient air pollution are likely to contribute to an increase in pollen allergies in the future [7,8].

Pollen monitoring networks have been developed in many countries to provide information to a wide range of end users such as allergy sufferers, researchers, pharmacists, and doctors [9]. An overview of pollen detection, measurement techniques, device development, and current networks is given by [10]. The most widely used method to monitor airborne pollen concentrations was developed in the 1950s and is based on manual microscopy analysis of collected samples using volumetric Hirst-type pollen traps [11]. Although considered as a standard method in aerobiology, this method is not only subject to a sizable range of uncertainties [12,13], but it is also time-consuming, labor-intensive, and expensive, making it impossible to have a dense surveillance network with a wide enough spatial coverage. Typically, only one instrument is operated in major cities or regions while a huge part of pollen spreads fall close to pollen emission sources, even if long-distance transport can also occur [14,15,16]. Moreover, pollen information is often available to the end user with a delay from one to several days from the related event, which goes against efficient health prevention and treatment strategies.

To tentatively automate the process of pollen monitoring and to provide end-users with an actionable and a higher quality information, various real-time pollen sensors have recently been developed. They involve physical concepts such as infrared spectroscopy, fluorescence spectroscopy, holography, image recognition, and the use of machine learning models [17,18,19,20,21]. Despite promising results when intercompared with Hirst-type traps [22,23], almost all these techniques still cannot meet the challenge of providing a local pollinic information with a high spatial resolution due to their significant cost, and the sensors are still requiring heavy calibration or maintenance operations.

A new automated and real-time pollen sensor named Beenose, developed by the Lify-Air company, could cope with these constraints, and could be deployed in dense networks. Beenose is based on light scattered by particles crossing a laser beam in the visible domain, which has been reported to help in distinguishing some pollen families from other ones in several studies [24,25,26,27,28,29,30]. The same principle has also been used to develop optical counters with typology determination capabilities, to distinguish between anthropogenic pollution particles, natural mineral particles, and pollens [31,32]. Beenose has already been tested in laboratory conditions, demonstrating its capacity to discriminate pollen particles from other particles and to characterize some pollen taxa [33].

Here, we first investigated the performance of Beenose in outdoor conditions. The sensor was deployed with a collocated Hirst-type trap in Belgium during the 2022 pollen season. We compared the consistency of the measurements in terms of daily average total pollen concentrations as well as daily average concentrations per pollen taxon. Next, we conducted a study on the spatial variability of the pollinic content of the air based on the measurements provided by four Beenose sensors deployed around Brussels at several kilometers apart, during a field campaign from mid-2022 to mid-2023.

The purpose of the paper is to conduct measurements on the pollen concentrations’ heterogeneity at a city level, and to highlight interests of developing a large network of new automated low-cost pollen sensors in supplementing historical methods, to better evaluate the real exposure of citizens to pollens.

## 2. Materials and Methods

### 2.1. Measurement Sites

**Figure 1:**
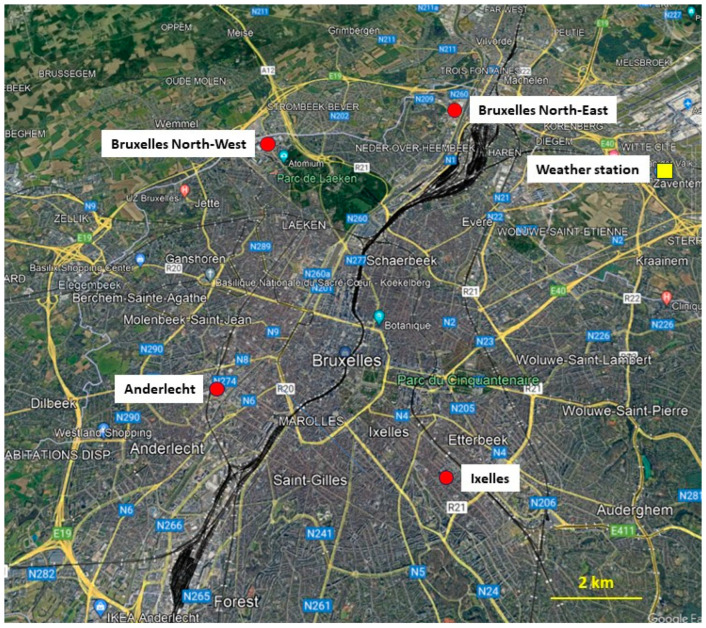
Measurement sites around Brussels, Belgium; north is up. Red dots represent the position of the sensors, and the yellow square represents the position of the weather station. Urban zones are in gray, parks and forests are in dark green, and landscapes are in pale green (map from Google Map).

The measurement campaigns were carried out in the Brussels-Capital Region area (Belgium). One Beenose sensor was installed at Ixelles next to a Hirst-type trap, while the three other Beenose sensors were installed in Anderlecht, Brussels North-West and Brussels North-East, respectively (Figure 1 and Table 1).

**Figure 2:**
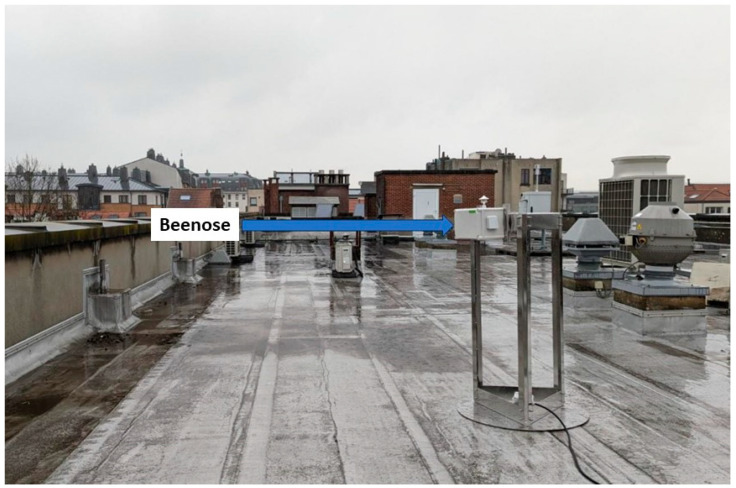
Beenose sensor at Ixelles, Belgium.

The experimental setup at Ixelles aims at evaluating the Beenose sensor performances. For this inter-comparison with the Hirst-type trap, both instruments were placed on the same building rooftop at a height of 14 m above ground level, 1.5 m above the rooftop level (Figure 2), and around 15 m from each other.

### 2.2. Beenose Sensor

The Beenose instrument works as an optical aerosol counter, based on the LOAC instrument design with measurements conducted at specific scattering angles [31]. Using a 360° inlet, the ambient air flow is pumped through an optical chamber at a flow of about 15 L·min^−1^, and the particles individually cross a laser beam. The scattered light is collected by optical detectors at 4 different scattering angles, 15°, 60°, 125°, 160°, which allows to partly describe the scattering curves and to retrieve some physical properties of the particles [25]. The measurements at the first angle are almost insensitive to the refractive index of the particles when they are not perfect spheres, thus a direct relation between particle size and scattering intensity can be established during laboratory calibration [34]. The measurements at the other angles are very sensitive to the shape and the refractive index of the particles and can be specific of the detected sample.

The intensities of the light signals are combined to retrieve the particle concentrations and typology (pollen families) for 19 sizes classes in the 5–100 µm size range. The typology identification is conducted using the “speciation indices” method [31], from the combination of the 4 channels’ measurements. A classification algorithm pretrained on the speciation indices identifies the samples that better match the laboratory measurements of the main pollen families and of ambient air pollutants [33]. Note that the scattering properties of an individual grain can differ from those of another grain of the same family, because of its orientation and rotation when crossing the laser beam. Thus, at least ten particles of the same nature and same size are necessary to retrieve the mean scattering properties of such irregular compact particles [35].

The software, covered by the intellectual property of the Lify-Air company that produces the Beenose instrument (Lify-Air SAS, Orleans, France), combines a first step of data cleaning, a second step of pollen/no pollen identification from the scattering properties of the particles, and a third step dealing with the taxa identification [33].

Beenose sensors are calibrated during the production process. Every sensor that presents a standard deviation of more than 5% in number concentrations in comparison with a laboratory reference Beenose sensor is immediately rejected from the production line. Regarding the efficiency of the collecting device and the mean concentrations, an uncertainty of about 20% is generally observed for Beenose sensors. Finally, the software version used in this study is the beta version developed by the Lify-Air company and takes account of the main pollen families in western Europe. The preprocessing and prediction algorithms were implemented using python libraries (pandas, numpy, scikit-learn) and are deployed on Amazon Web Services for online detection.

When the concentrations are very high, of about 1000 particules·m^−3^ (varying a little depending on the particle typologies and sizes), Beenose is not able to count all the particles, as a classical issue with automatic optical detectors [36]; in this case, the concentrations may be underestimated. As the purpose of the Beenose sensor is an end-user focused approach, based on informing the allergy sufferers of a potential risk, this issue is not critical for this application since this problem appears far over the concentration of pollens having an impact on human health.

### 2.3. Hirst-Type Trap

Hirst-type traps are volumetric samplers that were first designed by Hirst in the 1950s [11] and are still the most commonly used instruments by pollen monitoring networks worldwide. The measurement principle involves collecting pollen grains by impaction on a sticky surface then counting them using a microscope. More precisely, the sampler is equipped with a drum which is coated with an adhesive plastic tape. The drum moves clockwise at rate of 2 mm/h and airborne particles including pollen grains are drawn up into the orifice of the instrument with a flow rate of 10 L·min^−1^ before they are impacted on the adhesive tape. A rotating wind vane installed on the top of the sampler ensures that the orifice is well oriented. The adhesive tape is removed in general 7 days later and is then analyzed under microscope after the application of a staining solution that reveals only pollen grains and spores.

Hirst traps offer a standardized method for pollen monitoring [37], allowing for consistent sampling across different locations and time periods. This facilitates comparison and analysis of pollen data collected from various sources. However, pollen concentrations recorded by these traps can be in some cases subject to significant errors as reported by [13,37,38], typically from 20% for concentrations greater than 100 particules·m^−3^, to 150% for concentrations of 1 particule·m^−3^. Then, we should speak of inter-comparison instead of comparison to absolute values when comparing Hirst measurements to those from another instrument.

For evaluation purposes and before conducting our study on pollen variability using Beenose sensors, a Hirst-type sampler [39] was operated as a reference in parallel with the Beenose sensor located at Ixelles. Hirst concentrations have been made available by the Belgian aerobiological surveillance network of Sciensano [40], which performed the measurements following the methodological guidelines of the European Aerobiology Society (EAS) and provided pollen concentrations for species monitored. Hirst total pollen concentrations were then obtained by summing all the counts realized for each individual taxon.

### 2.4. Statistical Analysis

To confirm that the Beenose instrument can be used for pollen studies, it is necessary to investigate the consistency between the Beenose sensor and the Hirst-type trap. The Beenose sensor located at Ixelles was in a testing phase during the 6 first months of the 2022 pollen season. Some common breakdowns such as power failure, electromagnetic disturbances, or contamination of the optical chamber occurred, leading to transient and random measurements, leading to about 8% of corrupted data.

Hirst concentrations were available on a daily basis; a preprocessing step was therefore necessary to conduct the comparison by converting Beenose concentrations into daily means (obtained, for each of the instruments, by averaging all the values available in the same day). The following statistical indicators were then selected to achieve the comparison:

- Pearson correlation coefficient: This coefficient measures the linearity of the relationship between two variables. Its use suggests that the variables come more or less from a normal distribution.
- Spearman correlation coefficient: This coefficient is a non-parametric measure of the similarity between two sets of values, and assesses whether the relationship between two variables can be described by a monotonic function regardless of any knowledge about the parameters of their distribution.

## 3. Results

### 3.1. Comparison with the Hirst Method

#### 3.1.1. Total Pollen Concentrations

**Figure 3:**
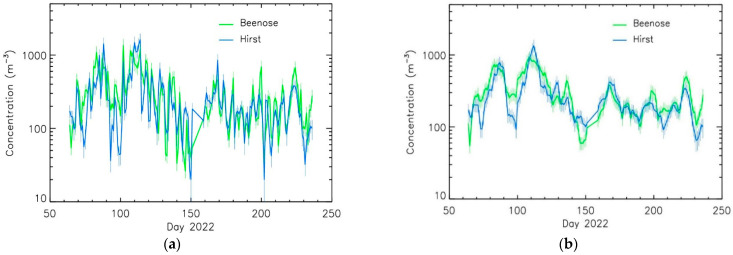
Comparison of daily total pollen concentrations between Beenose and the Hirst method, at Ixelles site: (**a**) raw values; (**b**) daily concentrations with a sliding smoothing window of 5 days.

The daily evolution of log-scaled average total pollen concentrations provided by the two instruments is shown in Figure 3a. Overall, the Beenose sensor is in line with the Hirst-type trap for both low and high total pollen concentration values, typically from 10 to 1000 particules·m^−3^, showing the same variations even if the former is limited by the counting saturation for high concentrations. A sliding smoothing filter of 5 days was applied for visual validation, showing that the Beenose instrument has well captured the main temporal evolution of the pollinic content provided by the Hirst sensor (Figure 3b). The annual pollen integral shows a mean error of 13% for the entire season. In case of perfect agreement, the correlation coefficients must be equal to 1; values greater than 0.5 indicate a good correlation. The good agreement between the two sensors’ measurements is confirmed by the similarity and the high values of the correlation coefficients: Spearman correlation coefficient of 0.71 and Pearson correlation coefficient of 0.72 (although the variables do not come from a perfect normal distribution). The values for the two correlation coefficients is above 0.80 when considering the 5-day average smoothing, used to remove the daily fluctuation from the main temporal tendency. These correlation values are similar to those obtained by other automated sensors [22].

**Figure 4:**
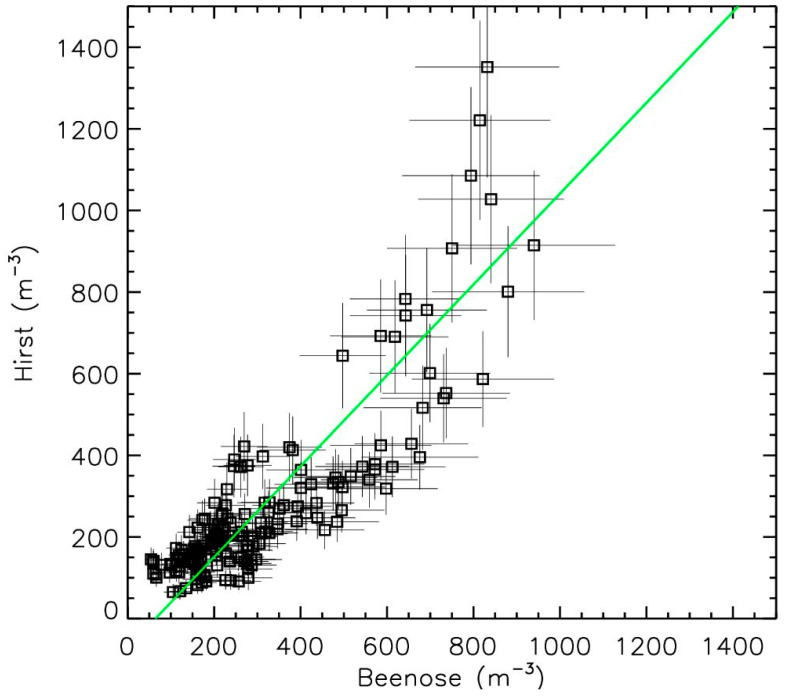
Correlation plot between Beenose (squares) and Hirst (linear fit in green).

The correlation plot (Figure 4) between the two sets of measurements shows a relationship close to 1 with an origin close to zero and overlapping error bars in most of the cases. The three highest values from Hirst measurements that are underestimated by Beenose are likely due to the saturation effect of this instrument. These results confirm the ability of Beenose to be used for the present scientific analysis of the pollen content. Obviously, other comparative studies should be conducted in the future for different locations and different taxa.

#### 3.1.2. Taxon-Specific Concentrations

Regarding the inter-comparison of concentrations for specific pollen species, six taxa were selected: Betula (birch), Alnus (alder), Corylus (hazel), Quercus (oak), Poaceae (grasses), and Urticaceae (nettles). Our study is limited to these species as they were the most critical and/or abundant ones during the studied seasons in the Brussels area among those detected by Beenose. Note that the first three taxa were grouped into one category as they belong to the same family (i.e., Betulaceae).

**Figure 5:**
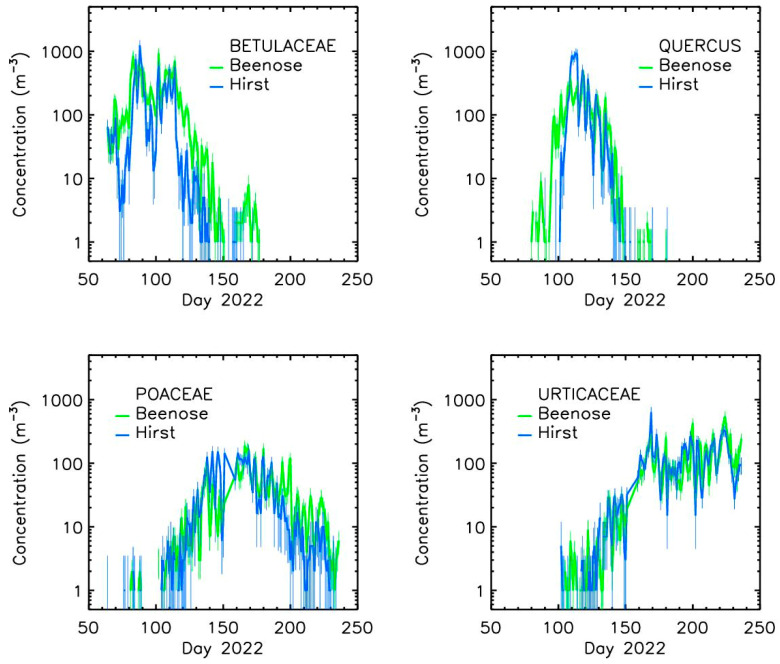
Comparison of the daily mean concentrations between Beenose and the Hirst method, from 4 pollen taxa, at Ixelles site.

Overall, the pollen species concentrations delivered by the Beenose sensor are in good agreement with those of the Hirst-type trap, all the main features being captured (Figure 5).

Table 2 shows the correlation coefficients for each pollen species and for total pollen concentrations. The Pearson correlation coefficients for Betulaceae, Quercus, Poaceae, and Urticaceae are 0.74, 0.72, 0.71, and 0.78, respectively. The Spearman correlation coefficients range from 0.81 to 0.93 depending on the species. As for the total concentrations, the consistency between the two sets of correlation values indicates that the typology identifications with the Beenose sensor provide usable results. The relatively small differences between Beenose and Hirst results could be explained by misclassifications of the Beenose sensor and by sampling issues for both instruments, mainly when concentrations are low. The saturation effect discussed for total concentration is another explanation.

### 3.2. Spatial Variability of Airborne Pollen Concentrations at Local Scale

**Figure 6:**
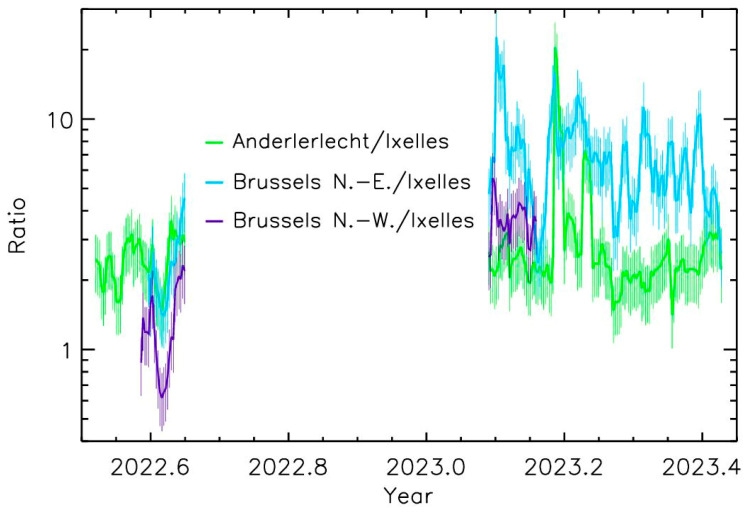
Time–evolution of the ratio of pollen concentrations measurements for the 3 sites to Ixelles concentrations measurements.

Four Beenose sensors were deployed in Brussels covering different conditions of local vegetation, city topology, and topographical conditions (Figure 1). The daily average concentrations of total pollen are here considered. The measurements encompass the middle of the 2022 pollen season and the beginning of the 2023 pollen season, for a total of 6 months. Figure 6 presents the time–evolution of the total pollen concentrations ratios for the three sites in comparison with the Beenose located at the reference site in Ixelles. Although the trends are almost similar, the absolute values differ strongly. The values at Ixelles are almost always the lowest ones. The amplitude of pollen concentrations depends on the stage of the pollen season for the two other sites: The highest concentrations are observed at Anderlecht and at Brussels North-East during summer, and at Brussels North-East for the beginning of 2023 where values reach up to about 10 times those recorded at Ixelles.

Such variability could be due to different factors such as elevation of the sensors, proximity of sources, and meteorological conditions. The pollen transport is a combination of winds’ strength and gravity (and perhaps of electric field if the particles are charged). Humidity can play a role only in changing a little the mass of the particles, but rain is also known for its action of pressing pollen to the ground.

**Figure 7:**
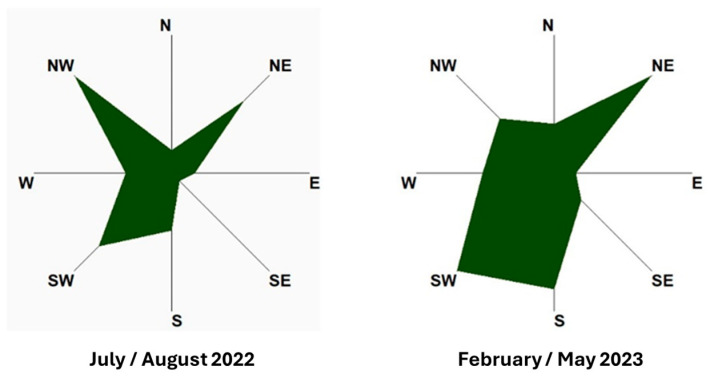
Weather compass rose at Brussels for the two periods of measurements (meteorological data from the Zaventem weather station). The scale of the radial axis provides the probability of the wind direction (the longer the shape in each direction, the higher the probability).

The meteorological data from the Zaventem weather station (50°53′ N, 4°28′ E), close to the Brussels airport and at 8 km from the center of Brussels in the north-east direction, are used to evaluate the effect of the wind on the pollens’ transport. For the two periods, the winds are mainly coming from the south to the north-west direction during the summer of 2022 (thus from the North Sea), and of less importance from the north-east direction (Figure 7).

**Figure 8:**
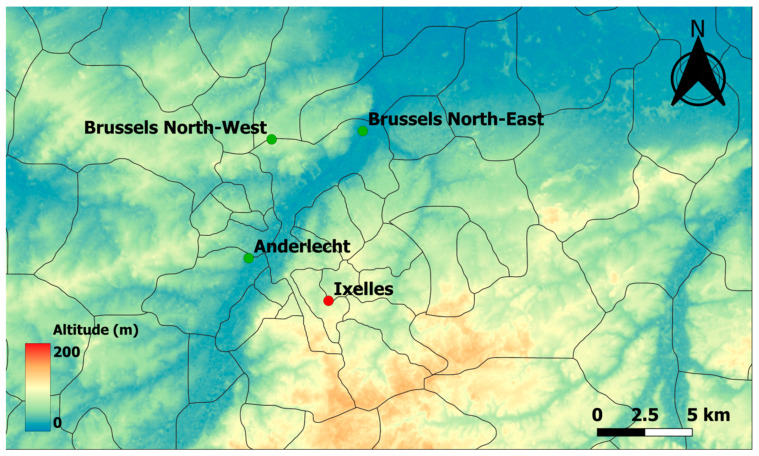
Altitude of the 4 sites; north is up.

As shown in Figure 1, the Ixelles site is in the middle of high-density urban houses and buildings, with forests and parks at several km in the south/south-east direction, indeed not exactly under the main wind directions. On the other hand, the Brussels North-East station is located at 3 km from a main park (Laeken Park), which is in the south-west direction of the measurement site and thus under the main wind direction (Figure 8). There are also crop fields at 2 km in the west north-west direction, under the main wind directions. The Brussels North-West station is close to the Laeken Park, but the park is in the opposite of the main wind directions and at a lower altitude than the sensor’s location. However, crop fields are present less than 2 km away with a partial exposure to the main wind directions. Finally, the Anderlecht site is in-between these situations, with some small parks 2 km away and agricultural fields at about 4 km in the west direction, under the main wind directions.

Finally, this analysis must be moderated by the fact that the sensors are not placed at the same altitude (Figure 8 and Table 1), including both the ground altitude and the height of the roof building where the instruments were installed. This is an important point when comparing absolute values [41]. The Ixelles and the Brussels North-West sensors are located on hills and are about 60 m higher than the two other stations. Thus, sources, wind transport, and elevation (altitude and height above the ground) strongly affect the pollen concentrations up to a factor 5 or more sometimes between two sites located only few kilometers apart.

## 4. Discussion

Considering the proximity of the sources, the main wind direction and the sensor elevations, some spatial trends could be qualitatively proposed. The concentrations in urban conditions (Ixelles) relatively far from the sources of emission or not under the main wind directions are always lower than those closer to the sources (Figure 6), even with distances of only a few kilometers. During the grass season (summer 2022), the three other sites compared to Ixelles exhibit relatively similar values. The situation is different when pollens originate from trees (here, the first part of 2023). Although the values are still often the lowest ones for the Ixelles sensor, the Brussels North-East sensor, downwind of a main park, exhibits the highest level of pollen concentrations. The two other sites (Anderlecht and Brussels North-West) located in areas with a moderate presence of trees exhibit lower values. Note that the short-time variabilities of the ratios over a few days at the beginning of March could be due to changes in the winds’ strengths and directions. This first analysis shows the spatial variability in pollen content to which the citizens are exposed.

Several studies address the need for a better understanding and thus modeling the pollen dispersion and transport from the local scale [42,43], with a strong concentration decrease over the first hundreds of meters, to the regional scale and beyond [44,45,46,47]. Pollen transport is a competition between gravitational settling and wind transport. The effect of height and altitude is thus also an essential parameter. A field campaign carried out in Thessaloniki, Greece has demonstrated that the pollen concentration could decrease in average by a factor 2 every 19 m of height [48]. Such variation depends on the pollen’s nature, as the larger pollen grains are the more difficult to be wind-transported while the smallest pollen grains easily reach a rooftop sensor. Qualitatively, the Beenose measurements seem to indicate such concentration evolution with altitude and height, but decorrelating the effect of these parameters from the effect of wind direction, sources’ location, pollens’ nature, and local topology is not an easy task.

The high variation of pollen concentrations in urban conditions detected around Brussels over a few kilometers is somehow similar to the one detected in Paris, France for the pollution particulate matter smaller than 2.5 µm (PM2.5) [49]. This study was conducted using measurements of hundreds of mobile sensors on cars to map in real time the city for which the local pollution sources are transport and heating. As for pollens, a factor 5 of variation was often found over several hundred meters due to the strength of the local sources, the wind transport, and the local topology. Such high variability must be considered when evaluating the local health effect of the population exposure to the PM2.5 pollution.

This analogy with PM2.5 pollution maps shows the necessity of conducting a large number of measurements in the same city to better evaluate in real time the local exposure of population to pollens, since allergy symptoms are directly related to the strength of pollen concentrations over time, which can vary in only a few days, and even more in only a few hours. At present, pollen monitoring is based at best on a single manual measuring station per city, which does not allow evaluation of the high variability of pollen concentrations locally, and thus to provide accurate pollen level alerts in real time. A dense network of real-time automated pollen sensors could be of great benefit to allergy sufferers to adapt their behavior regarding their own real-time micro-scale location. Also, such networks could help in pollen transport modeling at the micro-scale and open up avenues of research to study the effect of the wind direction and velocity, the effect of the height, the altitude, and the local topology on pollen concentrations, and to better understand phenomena such as the canyon street effect [50] that can trap the particles and then increase locally the concentration levels. Such study may grant new insights to the field of urban design and planning of green spaces.

## 5. Conclusions

The comparison of the Beenose sensor with the Hirst-type trap demonstrates that the measurements of the two instruments are consistent enough to conduct scientific studies with Beenose (even if the sensor outputs should be further improved with new software development in order to yield more consistency and robustness in all conditions).

The 6-month measurements of the four Beenose sensors around Brussels have shown the spatial variabilities of the pollens’ content at a local scale, which depends on the period of the year, the pollen natures and origins, the location in the city, the altitude, the height from the ground, the proximity to the emitting sources, and the wind direction. A local variation of pollen concentrations by typically a factor 5 or more can occur over a few kilometers. Such variability must be considered for allergy management strategies. Thus, the recommendations related to the Hirst-type traps’ installation (rooftops, far from pollen sources) lead probably to underestimate the authentic human exposure to the aeroallergens at the ground level, which could have detrimental effects for the management of allergy diseases.

These first results show the interest of developing, as for high-spatial PM2.5 pollution mapping, a network of hundreds of relatively low-cost and real-time pollen sensors. Following the experience on local PM pollution variability, at least one sensor per one to 5 km^2^ could be a good first objective. Obviously, more sensors should surround the main parks. Providing such data could allow the allergy sufferers to better adapt their treatment in response to the real pollen concentrations. Also, such future large sets of data could help to strongly improve the modeling calculations on pollen transport, and thus to provide better concentration values for locations where measurements are not available.

Further studies might focus on (i) the evaluation of the number of micro-sensors that would be needed to efficiently cover an area of 30 km in diameter, (ii) the advantages or disadvantages of combined networks of micro-sensors and Hirst-type traps that are validated at a larger territorial scale, and (iii) the potential of the micro-sensors to deliver relevant hourly pollen information that should help allergy sufferers to adapt their behavior with eviction measures and treatments.

## Figures and Tables

**Table 1:** Campaign sites.

Location	Coordinates	Altitude (m)	Height above the Ground (m)
Anderlecht	50°50′40.6″ N 4°19′24.8″ E	16	9
Brussels North-West	50°54′02.1″ N 4°20′26.4″ E	83	11
Brussels North-East	50°54′16.3″ N 4°24′31.3″ E	24	9
Ixelles	50°49′28.3″ N 4°22′59.3″ E	84	14

**Table 2:** Summary of the statistical metrics calculated for the total pollen concentrations and the taxon-specific concentrations.

Taxon	Spearman Correlation Coefficient	Pearson Correlation Coefficient
Betulaceae	0.86	0.74
Quercus	0.81	0.72
Poaceae	0.88	0.71
Urticaceae	0.93	0.78
Total taxa	0.72	0.71

## Data Availability

The data are the property of the Lify-Air Company, and the Hirst pollen data are the property of Sciensano, but can be provided under request for scientific purposes only.
